# Novel Concept to Detect an Optimum Thixoforming Condition of Al-Al_3_Ni Functionally Graded Material by Wavelet Analysis for Online Operation

**DOI:** 10.3390/ma4122183

**Published:** 2011-12-14

**Authors:** Noriyoshi Kumazawa, Yasuyoshi Fukui, Daisaku Nara

**Affiliations:** Graduate School of Science and Engineering, Kagoshima University, 1-21-40, Korimoto, Kagoshima 890-0065, Japan; E-Mails: fukui@mech.kagoshima-u.ac.jp (Y.F.); nara@eng.kagoshima-u.ac.jp (D.N.)

**Keywords:** wavelet analysis, control, thixoforming, functionally graded material, intermetallic compound

## Abstract

A novel technique to characterize the transition phenomenon from solid to melt of Al-Al_3_Ni functionally graded material (FGM) through a wavelet analysis for the development of a thixoforming system is investigated. Identification of an optimum semi-solid condition for thixoforming is necessary not only for the construction of a system but also the fabrication of a near-net-shape product with fine microstructure. An online wavelet analysis system using Haar’s wavelet function, which is applied for its simplicity compared with Daubechies’ wavelet function, is developed to find the optimum operating condition. A thixoforming system, which is constructed adapting a threshold value as an index, monitors successfully a discontinuity of deformation of Al-Al_3_Ni FGM with the temperature rise. Thus, the timing of an operation is not at pre-fixed temperature but at the time when the index related to a wavelet function is satisfied. The concept is confirmed to be suitable from the micro-structural observation of the Al-Al_3_Ni FGM product, because the product under the optimum condition is found to have refined Al_3_Ni grains, which change from coarse grains and are expected to improve the mechanical properties.

## 1. Introduction 

Functionally graded material (FGM) possesses a unique character that it has a composition gradient in one direction and has no macroscopic boundary [1,2,3]. The present authors proposed one manufacturing method, named a centrifugal method, to make FGMs and reported experimental results using various FGMs fabricated by this method [4,5,6,7,8]. In particular, Al-Al_3_Ni FGM, which is a kind of metal-intermetallic compound FGM, has been developed as a structural and/or component material [5,7,8,9]. However, the FGM manufactured by the centrifugal method contains coarse grain of Al_3_Ni intermetallic compound, which is hard and brittle in nature, due to slow cooling rate [8,9]. Such coarse grains make the plastic forming impossible at cold working temperature and at hot working temperature even just below the melting point. Therefore, it is necessary to examine a proper forming method for the application of the Al-Al_3_Ni FGM to a practical usage. 

Al-Al_3_Ni FGM is regarded as a kind of eco-FGM because the FGM has been recycled repeatedly in the laboratory [10]. During the experimental process, it was found that the Al matrix is easily melted but the Al_3_Ni intermetallic compound is difficult to melt. Based on our previous studies [5,7,8,9], we have been led to an idea that a near-net-shape product with forged effect can be obtained if the FGM is formed under the condition of semi-solid aluminum matrix and solid Al_3_Ni particles. This idea may be regarded as an application of semi-solid forming [11,12,13,14] but the difference is that the material is inhomogeneous and the solid Al_3_Ni particles are dispersed in the material. Thus, semi-solid forming, *i.e*., thixoforming, is proposed as a near-net-shape forming process and studied in this paper. A determination of the optimum condition is necessary and useful to clarify the cause of the results. The experimental results show that Al_3_Ni particles of large size grains transfer to fine grains at processing temperature just upper the eutectic melting point [8]. There still remains the problem how to confirm both the semi-solid condition and operating index of the system only monitoring the temperature of die and/or billet.

It is well known that the wavelet analysis is one successful mathematical tool for analyzing a signal and has superior functions as same as the Fourier analysis. A time-frequency localization of unsteady signals has been analyzed by the wavelet analysis and a discontinuity of particular signals is detected through wavelet coefficients [15]. Recently, the wavelet analysis is applied to denoise of signals [16], image processing [17], data compression and communication network [18], system identification, numerical analysis of differential equations [19] and analysis of stress concentration [20]. All these applications come from the fact that the wavelet analysis is useful to detect the transition point of a signal [21]. In the present study, the flow stress of Al-Al_3_Ni FGM with heating up to dissolution show a transition from elasticity to visco-plasticity which corresponds to that from solid to semi-solid condition. Therefore, a discontinuity corresponding to the transition from solid to semi-solid condition can be detectable if the signal of flow stress or corresponding index, which is a function of temperature, is analyzed by wavelet analysis. As will be shown, the temperature just above the eutectic melting point may be an optimum operating condition.

In the following section, we proposed a control technique for thixoforming system which utilizes the wavelet analysis to detect an optimum forming temperature of an Al-Al_3_Ni FGM. An index of semi-solid state of the Al-Al_3_Ni FGM, which is evaluated from wavelet coefficients, is applied to a thixoforming system. A thixoforming of a backward extruding method is used to Al-Al_3_Ni FGMs for the experimental confirmation. Appropriateness of the constructed system is judged based on the microscopic observation of the Al-Al_3_Ni FGM product and the effectiveness of the proposed method is discussed.

## 2. Materials and Experimental Procedures

### 2.1. Preparation of Al-Al_3_Ni FGM Billet

**Figure 1:**
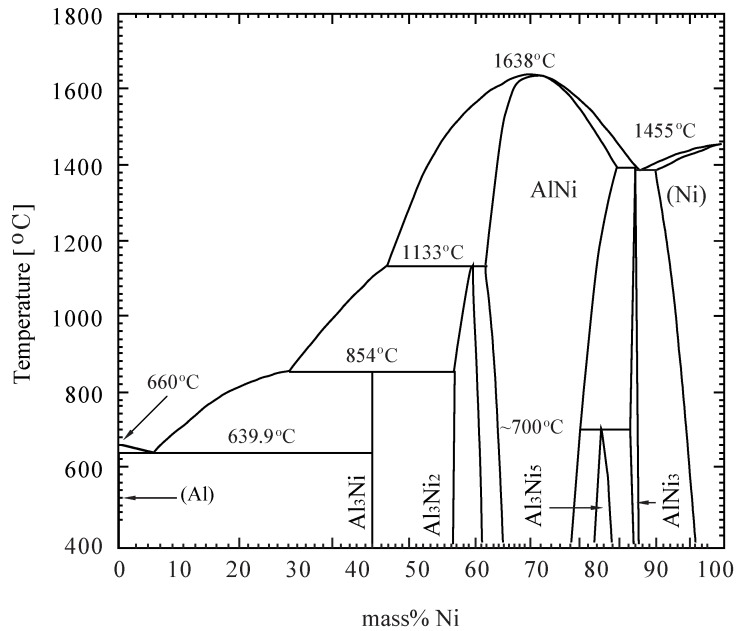
Al-Ni two phase diagram [22].

The master Al-Al_3_Ni FGM is fabricated by a centrifugal casting machine using commercial Al-20 mass% Ni alloy ingot which is melted at 830 °C under Ar gas atmosphere and then poured into the rotating mold. Figure 1 shows a binary phase diagram of Al-Ni system [22], and the melting point of Al-20 mass% Ni alloy is about 805 °C. The FGM product is thick-walled tube and the gradation of the volume fraction of Al_3_Ni intermetallic compound is in the range from 50 vol% at outside of the tube to 0 vol%, *i.e*., pure Al, at inside. A backward extruding is applied to the Al-Al_3_Ni FGM billet for the manufacturing of near-net-shape product of a simple FGM cup. FGM billet of 38 mm diameter 15 mm thickness for a backward extruding is cut out by the electro-discharge machine from the FGM tube such that the thickness direction coincides with the radial direction. Figure 1 indicates that the present Al-Al_3_Ni FGM billet begins to melt at 639.9 °C and Al_3_Ni grain of the line compound is stable upto 854 °C. The semi-solid condition of the billet controls by temperature, heating rate, heating time.

### 2.2. Setup for Near-Net-Shape Forming

**Figure 2:**
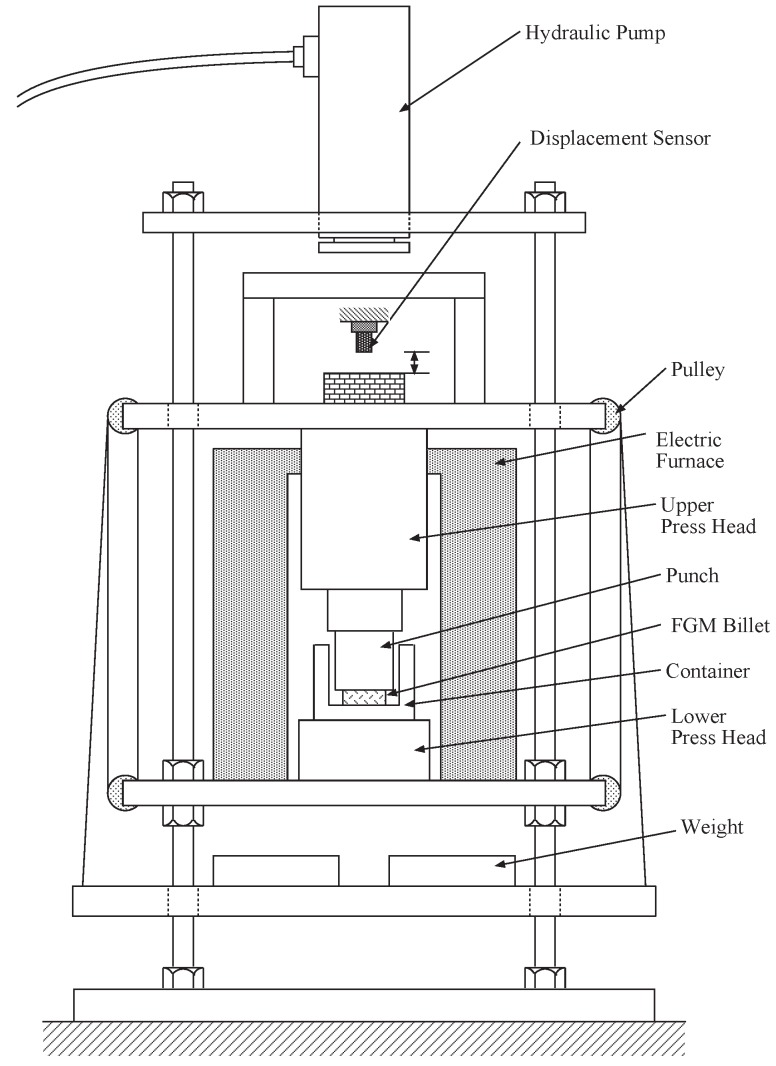
Schematic representation of functionally graded material (FGM) semisolid forming system.

**Figure 3:**
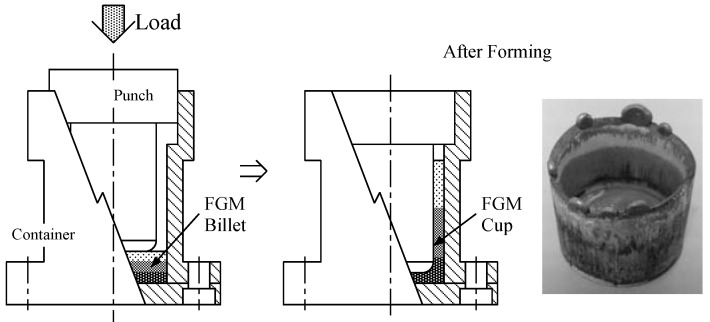
Schematic illustration for FGM semisolid forming process.

The machine for thixoforming constructed in the present study is illustrated in Figure 2. It consists of an electric furnace, press head, die, displacement sensor, hydraulic pump, *etc*. An outline of backward extruding method applied to the FGM billet in the die is presented in Figure 3. The die consists of punch and a container in which FGM billet of aluminum rich surface contacts with punch. The die and billet set for the backward extruding is heated in an electric furnace and then a pressing load is applied at some index temperature as shown in Figure 3. The temperature of the billet is controlled by the output of a thermocouple inserted into the bottom of the container. Finally, the billet is deformed in the container as shown in Figure 3 and an FGM cup of 38 mm outer diameter × 40 mm height × 4 mm bottom thickness × 3 mm wall thickness is obtained. 

Flow stress relates directly to the axial deformation of the billet which is yielded from heating under constant applied load. Thus the deformation may be utilized instead of flow stress to analyze the optimum forming condition. The upper press head is loaded by a dead load of 3 kN applied to the punch in order to monitor the axial deformation of the billet with temperature increase. A displacement sensor is set above the upper press head. The value of 3 kN is decided to take the variation of the flow stress of Al-Al_3_Ni FGM at hot working temperature into account to avoid a meaningless deformation which prevents the analysis. The difficulty to detect the transformation of FGM billet from solid to semi-solid condition in the container must be overcome to analyze the output of displacement. The signals of displacement as a function of time are analyzed by the wavelet function introduced to thixoforming system. The analyzed condition is feed back to the machine to start the forming operation.

## 3. Application of Wavelet Analysis

### 3.1. Haar’s Wavelet

For the control of system, it is necessary to introduce the wavelet analysis to the output signals measured from a displacement sensor mounted on the machine as shown in Figure 2. The wavelet transformation is defined as follows [23]:

(1) $$d_{k}^{\left(j\right)}\;:\;=2^{j/2}\int_{-\infty }^{\infty }\bar{\psi (2^{j}x-k)}\;f(x)\;dx\;(j=0,\;1,\;2,\ldots ,\;k=0,\;1,\;2,\;\ldots )$$

where f(x) is a function of the analyzed signal, and ψ is a basis function called mother wavelet. An index j is a level of resolution and k is a shift parameter. The signal f(x) is approximated to:

(2) $$c_{k}^{\left(j\right)}=2^{j}\int_{k/2^{j}}^{(k+1)/2^{j}}f(x)\;dx$$

by adopting the following step-formed function:

(3) $${f_{j}(x)\;|}_{\;I_{k}^{\left(j\right)}}=c_{k}^{\left(j\right)}$$

where the intervals are given by:

(4) $$I_{k}^{\left(j\right)}=\left[k/2^{\left(j\right)},\;(k+1)/2^{\left(j\right)}\right]\;(k=0,\;1,\;\cdots ,\;2^{\left(j\right)}-1)$$

In the present study, the well-known Haar's wavelet, which is often applied to analyze a time-frequency localization, is used and is written as follows:

(5) $$\Psi_{H}(\tau )=\left\{ \begin{array}{l} 1,0\leq \tau \leq \frac{1}{2}\\ -1,\frac{1}{2}\leq \tau \leq 1\\ 0,otherwise \end{array}\right.$$

Then wavelet coefficient of level j, $g_{j}$, is introduced as the difference of step function between a level j and a level j+1. $g_{j}$ is a time-dependent function and is written as follows:

(6) $$g_{j}=\sum_{k}d_{k}^{\left(j\right)}\;\psi_{H}(2^{j}x-k)$$

where $d_{k}^{\left(j\right)}$ is given as follows:

(7) $$d_{k}^{\left(j\right)}=\frac{1}{2}\left(c_{2k}^{\left(j+1\right)}-c_{2k+1}^{\left(j+1\right)}\right)$$

### 3.2. Detecting a Inflection Point of Signal

**Figure 4:**
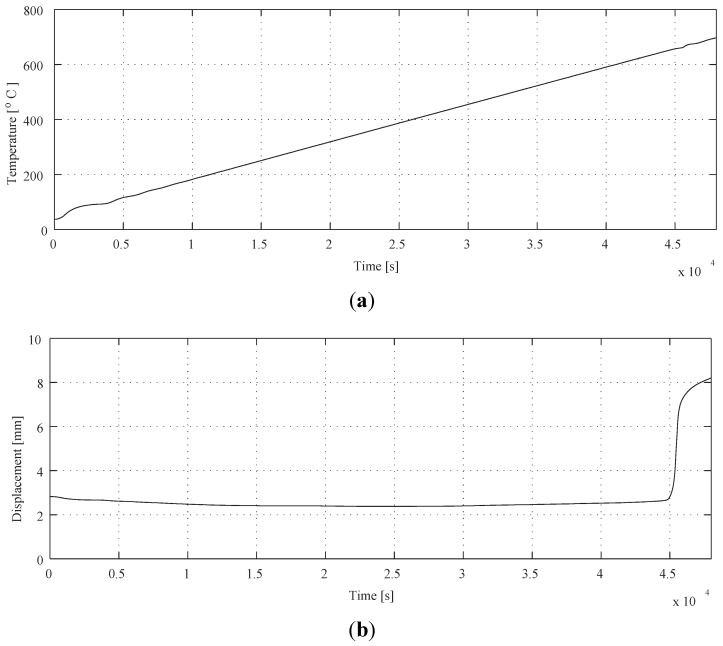
(**a**) Controlled FGM billet temperature; (**b**) displacement of FGM Billet.

A preliminary experimental results of the variation of temperature and axial displacement as a function of the time are given in Figure 4. The sampling time is 1 s and the die is heated from ambient temperature to 700 °C by the heating rate of 50 °C/h. The displacement measured by an eddy current sensor, which is set unconnected to the body of the machine, arises from the heating of billet under the applied compression dead load of 3 kN. The 3 kN load is enough to detect the visco-plastic deformation of the present size billet. On the other hand, the temperature of the FGM billet is monitored by a thermocouple inserted in the bottom of container. The temperature is regulated by the PID controller of which design parameters are tuned by the critical gain method [24]. A constant heating rate is applied to avoid the ambiguity of heating curve, *i.e*., having no transition point. The heating rate is decided from the consideration of the performance of both electric furnace and the control law.

**Figure 5:**
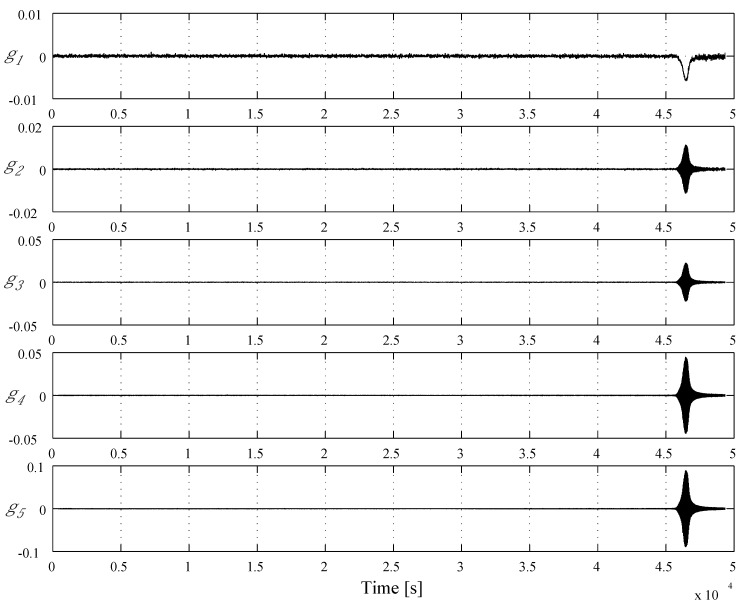
Result of the wavelet analysis (Haar).

The variation of temperature as a function of time of Figure 4(a) shows smooth linear relation as expected. However, the displacement curve is complicated as shown in Figure 4(b). Thermal strain, *i.e*., displacement, is decreased until 2.5 × 10^4^ s and then turned to increase. This is because the output of the sensor contains not only compressive deformation under applied dead load of 3 kN but also the thermal expansion of the body of the machine. The latter governs the first 2.5 × 10^4^ s in Figure 4(b) and then the former controls a profile of the curve after that. The abrupt increase of thermal strain begins at around 4.5 × 10^4^ s and is attributed to the reduction in billet height associated with a transition from solids to semi-melt under application of dead load. The character of thermal expansion is known as steady state deformation that the effect can be ignored even if it is included in the signal. Thus, the signal of measured thermal strain shown in Figure 4(b) is analyzed by the Haar's wavelet and the result is shown in Figure 5. The calculated results of sequential processing, $g_{i}$, i=1,2,...,5, are shown in order of up($g_{1}$) to down($g_{5}$). It can be easily seen in Figure 5 that an inflection point of the displacement curve, which is caused by a meltdown of the FGM billet, is analyzed by variation of wavelet coefficients at about 4.6 × 10^4^ s.

### 3.3. Determination of Threshold Value

In case of wavelet analysis, it is known that component parts which are related to the vanishing moment’s property can be detected. By reason of that, the results of Figure 5 by Harr’s wavelet indicate the components of the signals related to strain rate. To find the real inflection points in a relationship between thermal strain and temperature, it is neessary to use a wavelet with a first order of vanishing moment of zero. The condition is written as:

(8) $$\int_{-\infty }^{\infty }x\;\psi (x)\;dx=0$$

**Figure 6:**
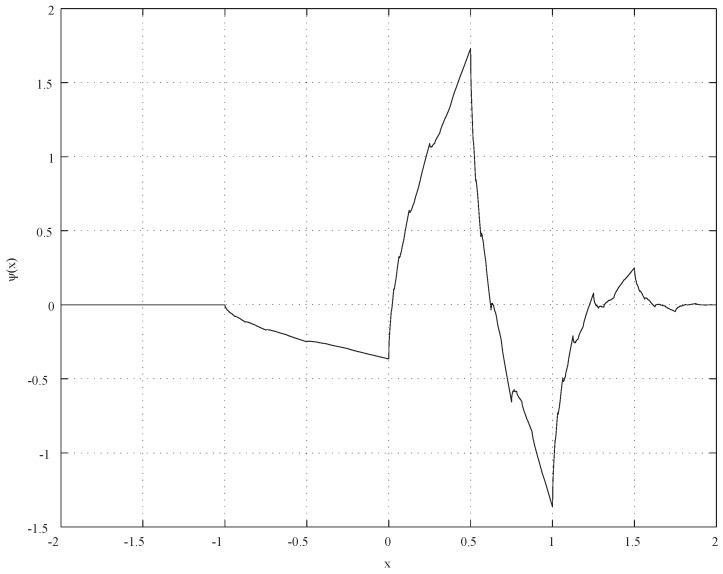
Daubechies wavelet (N = 2).

**Figure 7:**
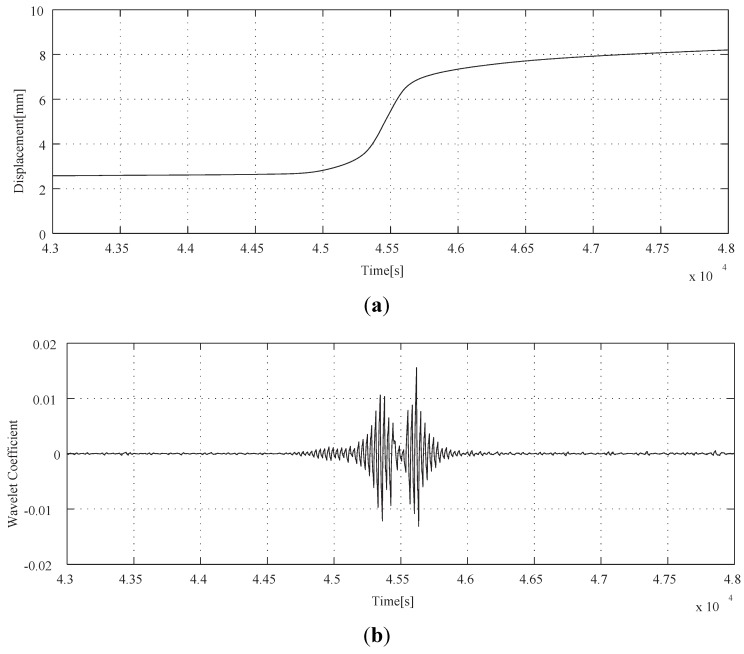
Result of the wavelet analysis (Daubechies). (**a**) Displacement of FGM billet; (**b**) Wavelet Coefficient to Displacement.

Thus, we introduce Daubechies’ wavelet as a scaling function that satisfies a momentum condition for analyzing displacement curves. The mother wavelets of Daubechies are indexed by N=2,3,... [25] and the wavelet _2_*ψ* shown in Figure 6 is used in this study. The derived function of _2_*g*_5_ is shown in Figure 7 which denotes three discontinuous points related to elasto-plastic and visco-plastic deformation. The transition point at 4.50 × 10^4^ s is corresponding to the temperature of Al-Al_3_Ni alloy eutectic melting point. Therefore, the method can detect a transition point from elasto-plastic to visco-plastic property of the Al-Al_3_Ni FGM. The result of Daubechies’ wavelet is the same as Haar’s wavelet. Thus, Haar’s wavelet is applied to the analysis of the manufacturing system in the present study, because the decomposition of signals using Haar’s wavelet can be coded more simply than that of Daubechies _2_*g*_5_.

Based on Figure 5, it is assumed that a value of wavelet coefficient $g_{5}$ at 4.50 × 10^4^ s is a threshold value to detect the semi-solid condition of Al-Al_3_Ni FGM. The definition of the threshold value ν is written as the form:

(9) $$\nu :\;=\left|g_{5}\right|$$

For the simplicity of analysis, the number of displacement data for each sampling time of 1 s is set in ${\left(2^{5}\right)}^{2}$ and the wavelet analysis is adapted to the data sampling time. The last value of the calculated time series of $g_{5}$ is decided as $g_{5}^{*}$ which is considered as a wavelet coefficient at that moment. The suitable forming condition is regarded as the coefficient $g_{5}^{*}$ firstly satisfies following relation:

(10) $$\left|g_{5}^{*}\right|\geq \nu$$

## 4. Operation of Thixoforming System

### 4.1. Process of Experiment

Since the forming starts at not pre-fixed temperature but at online calculated temperature based on the threshold value ν, it becomes important how to detect the starting temperature of thixoforming. Because the semi-solid condition depends not only on temperature but also heating rate and heating time, the value of $\left|g_{5}^{*}\right|$ calculated using wavelet analysis in Section 3.3, gives the condition to start the forming, namely the relation of Equation (10) is an index of a forming operation. When the condition is satisfied, a hydraulic pump starts to run automatically and the FGM billet in the container is compressed through the press head by a hydraulic press up to an established load of 100 kN.

The flow of the thixoforming process in this study is summarized as follows:

Step 0: Set a threshold value ν through a preliminary test.

Step 1: Heat the FGM billet continuously in a fixed rate as a ramp type.

Step 2: Calculate $g_{5}^{*}$ from the finite displacement data for each sampling time.

Step 3: Go Step2 if the Equation (10) is not satisfied.

Step 4: Apply a load of 100 kN through a hydraulic pump.

Step5: Remove a load and cool the die to ambient temperature.

Note that the heating rate in the Step1 must be the same in the Step 0. If a different heating rate is selected, another preliminary test is necessary to decide the corresponding threshold value.

### 4.2. Results and Discussions

**Figure 8:**
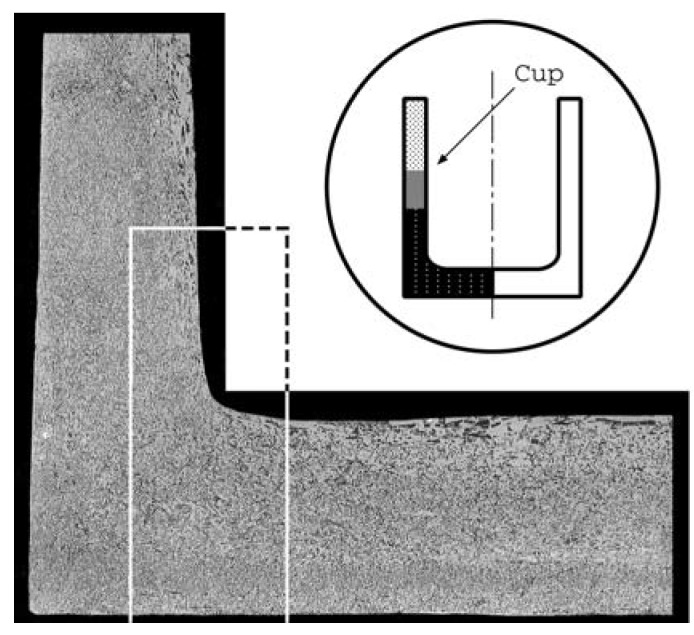
Micrographs showing the profile of the intermetallics in Al phase after forming.

**Figure 9:**
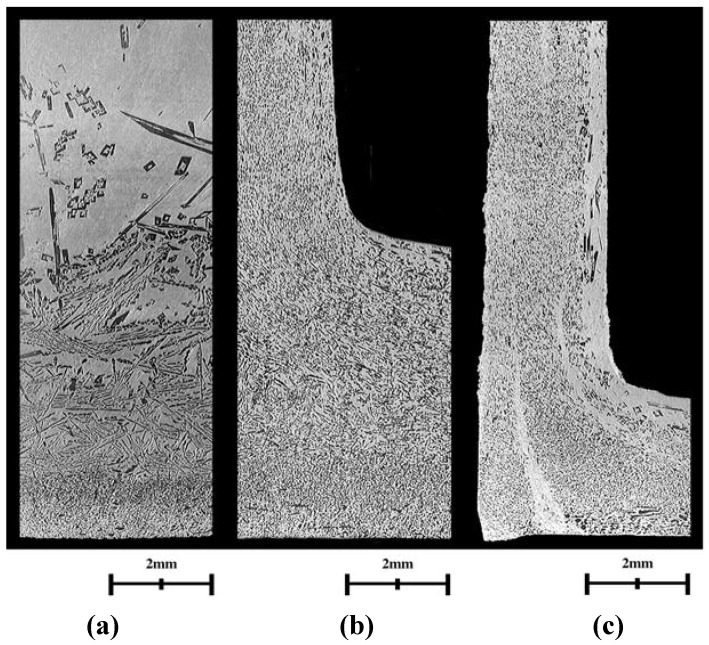
Higher magnification views showing the profile of the intermetallics. (**a**) Original profile; (**b**) Optimum result; (**c**) Poor result.

Metallographic observation of the backward extruded Al-Al_3_Ni FGM cup is done for the evaluation of effectiveness of the proposed system. Figure 8 shows the cross-section of a cup formed by the optimum process mentioned in section 4.1. Higher magnification views of Figure 9 from (a) to (c) show the original, optimum result and poor result for the comparison, respectively. In Figure 8 and Figure 9, the white part is Al matrix and dark particles are Al_3_Ni intermetallic compound. Figure 9(a) is the profile of the Al-Al_3_Ni FGM billet before forming and the shapes of Al_3_Ni particles are different depending on the position. They are roughly spindle, bar and granular shape at the upper, intermediate and lower region, respectively. Figure 9(b) is a higher magnification view at the position indicated in Figure 8 and shows that the distributed particles in Figure 9(a) are changed to be fine fibrous and/or rounded shape under an optimum operating condition. The result of the unsuitable setting of the larger threshold value ν, that means the adapted temperature is higher than the optimum condition, is shown in Figure 9(c). There exist lots of coarse Al_3_Ni particles in contrast to Figure 9(b). Moreover, macroscopic aluminum matrix flow lines are observed in Figure 9(c). This suggests that the molten metal does not hold a sufficient viscosity to produce fine Al_3_Ni particles. The result agrees with the former results [8] that lower operating temperature produces a better microstructure. The metallurgical process and/or mechanism to produce fine Al_3_Ni particles will be reported in near future. 

We have taken up the semi-solid forming to overcome the failings of the coarse grains of Al_3_Ni intermetallic compound in an Al-Al_3_Ni FGM, that make the plastic forming impossible at hot working temperature even just below the melting point. It is difficult to detect the optimum semi-solid state for forming directly from materials and then the process is usually done at a prefixed temperature. Referring to the lecture of Flemings [11], the semi-solid forming has to be done at solid and liquid coexistence temperature range considering the phase diagram. Many studies to evaluate the semi-solid state during processing have been reported. Ultrasonic, for example, is applied to monitor the condition of semi-solid metals during processing [26]. A process using acoustic emission to determine the semi-solid condition by induction heating [27] is also reported. These are a kind of response analysis and are independent of temperature measurements same as the present study to eliminate the ambiguity over the semi-solid condition. However, the viewpoint is somewhat different to an online detection of the optimum operation timing. Thus, the process studied here evaluates adequacy of the method based on a prefix index of semi-solid state of the Al-Al_3_Ni FGM through microstructural observation.

The proposed thixoforming system, which applies the wavelet analysis to detect the optimum operating condition, gives suitable formed products as shown in Figure 8. It is very easy and simple to adopt Harr’s wavelet to the developed system, even though the discontinuity of the thermal strain *vs.* temperature curve cannot be used positively. The reason of this is that the index corresponds to strain rate as mentioned in Section 3.3. To avoid the shortcoming, the strain rate is derived from low pass filtered signals of displacement differences for a relation among temperature, displacement and time shown in Figure 4 and a threshold value is calculated from the strain rate. The threshold value is applied to the thixoforming and similar good result is obtained. The problem is the difficulty to set a proper crossover frequency of a filter which excludes undesired signal time delay because the optimum value is found by a trial-and-error method. More proper condition must be obtained from Daubechies’ wavelet which satisfies the momentum condition of Equation (8) and is used for the scaling function of this study. Unfortunately, the decomposition of signals by Daubechies’ wavelet is a complicated procedure to adopt the present system. On the other hand, the proposed method using Haar’s wavelet is an effective tool to detect the semi-solid condition of the metallic material, *i.e*., thixoforming condition.

## 5. Summary

In this paper, the applicability of the wavelet analysis to detect the optimum thixoforming operation temperature of Al-Al_3_Ni FGM is investigated. A thixoforming system, which is adapted the wavelet analysis, is developed for the purpose. The confirmation of the semi-solid condition and operating index of the system is done through monitoring the signal of axial deformation of billet by heating. The wavelet coefficients, which are derived using Haar's wavelet, are calculated to decide an index of the semi-solid condition of material. The optimum operation timing related to the proposed index is established well through a continuous analysis of the signal for each sampling time. Then, Al-Al_3_Ni FGM cups are fabricated by a thixoforming of a backward extruding method. When the Al-Al_3_Ni FGMs were formed using the threshold value proposed in this study, Al_3_Ni particles in the FGM cup changed to be more fine particles are different from those in other poor condition. Thus, an availability of the proposed online thixoforming system, which applies the wavelet analysis for an active control, is confirmed.
